# Effectiveness of Arbidol for COVID-19 Prevention in Health Professionals

**DOI:** 10.3389/fpubh.2020.00249

**Published:** 2020-05-29

**Authors:** Chunguang Yang, Chunjin Ke, Daoyuan Yue, Wengang Li, Zhiquan Hu, Wei Liu, Shuhua Hu, Shaogang Wang, Jihong Liu

**Affiliations:** ^1^Department of Urology, Tongji Medical College, Tongji Hospital, Huazhong University of Science and Technology, Wuhan, China; ^2^Department of Clinical Laboratory, Tongji Medical College, Tongji Hospital, Huazhong University of Science and Technology, Wuhan, China; ^3^Department of Prevention and Health, Tongji Medical College, Tongji Hospital, Huazhong University of Science and Technology, Wuhan, China

**Keywords:** SARS-CoV-2, COVID-19, arbidol, health professional, primary prevention

## Abstract

**Background:** Frontline health professionals are a COVID-19-susceptible population during the outbreak of COVID-19, but prophylactic drugs against SARS-CoV-2 infection are to be explored.

**Method:** Frontline health professionals diagnosed with COVID-19 before February 9, 2020 in Tongji Hospital, Wuhan, China and the same amount of controls in the uninfected group were included in this study. Clinical and laboratory data were collected with standardized forms.

**Results:** A total of 164 subjects were included in this study, 82 cases in the infected group and 82 controls in the uninfected group, with a median age of 37 years, including 63 males and 101 females. Nineteen (23.2%) patients in the infected group were administered oral arbidol, and 48 (58.5%) in the uninfected group (OR = 0.214, 95% CI 0.109–0.420). The cumulative uninfected rate of health professionals in the arbidol group was significantly higher than that of individuals in the non-arbidol group (log-rank test, χ^2^ = 98.74; *P* < 0.001). Forty-eight patients (58.5%) in the infection group were hospitalized, with a median age of 39 (31–49) years, of whom 7 (14.6%) were prophylactically administered arbidol. Thirty-four patients (41.5%) with mild symptoms were treated outside the hospital, among which the median age was 34 (30–39) years, and twelve patients (35.3%) took prophylactic oral arbidol. The hospitalization rate was significantly associated with age (*P* = 0.024) and oral arbidol administration (OR = 0.313, 95% CI 0.108–0.909). In the age-matched case-control study, the hospitalization rate was not significantly associated with arbidol administration (*P* = 0.091).

**Conclusion:** Prophylactic oral arbidol was associated with a lower incidence of SARS-CoV-2 infection but not hospitalization rate in health professionals, providing a basis for the selection of prophylactic drugs for high-risk populations.

## Introduction

Coronavirus disease 2019 (COVID-19) has spread rapidly worldwide since its discovery in December 2019 (1). As of April 14, 2020, SARS-CoV-2 has affected a total of 1.8 million people, including tens of thousands of health professionals (2). Health professionals are susceptible to COVID-19. Previous literature confirms that the work area of health professionals significantly affects the probability of infection when they are in close contact with the coronavirus (3). Moreover, studies have shown a significant correlation between age and prognosis of patients infected by SARS-CoV-2 (4). However, currently, there are no preventative drugs supported by clinical research (5). Experiments have shown that arbidol, namely umifenovir, inhibit viral replication for SARS coronaviruses (6). Arbidol was also shown to block virus replication by inhibiting the fusion of influenza virus lipid membranes with host cells (7). Based on the results of the above studies and the availability of the drug, some health professionals in Tongji Hospital preventatively took the oral antiviral drug arbidol in clinical practice on themselves, but its role is not clear.

Therefore, we used an age-matched case-control study to retrospectively analyse the correlation between COVID-19 and preventative oral arbidol use among health professionals in Tongji Hospital to explore the impact of arbidol on COVID-19 among health professionals.

## Methods

### Study Design and Participants

After the outbreak in Wuhan, a large number of health professionals in our hospital were on the front line of the outbreak, which is a good sample for analysis. Therefore, in-service health professionals in Tongji Hospital diagnosed with COVID-19 by throat swab nucleic acid test (infection group) before February 9, 2020 were retrospectively selected. Based on age and work area, they were frequency matched, and the same number of uninfected health professionals working in Tongji Hospital (uninfected group) was selected. High-risk departments included outpatient and emergency departments, the fever ward, the respiratory department, thoracic surgery, and the infection department, whereas the other departments are non-high-risk departments. Whether the infected and uninfected cases were prophylactically administered oral arbidol before being selected is unknown. The protective measures adopted by the health professionals were unanimously requested in the same department or work area, such as protective suit, goggles, masks, etc. Patients in the infection group who took arbidol within 2 weeks before the first symptom were defined as taking arbidol. Subjects in the uninfected group who took oral arbidol during the same period were also defined as taking arbidol. The preventative dosage was defined as 200 mg qd po, whereas the therapeutic dosage was defined as 600 mg qd po.

This study was reviewed and approved by the Medical Ethical Committee of Tongji Hospital of Huazhong University of Science and Technology (IRB ID:TJ-C20200133).

### Data Collection

Information collection was accomplished mainly through our hospital's electronic medical record system and telephone interviews. The data collection indicators included mainly the subject's age, sex, comorbidities, occupation, work department, COVID-19 onset time, arbidol administration, isolation location (hospital/home/hotel), laboratory parameters, present of severe pneumonia during hospitalization and clinical outcomes. Clinical were obtained with standardized forms for all subjects involved. Two researchers independently reviewed the data.

## Outcomes

The distribution of COVID-19 among health professionals in our hospital since Jan 5, 2020 was determined. Statistical analysis was included to study the relationship between baseline characteristics of health professionals and SARS-CoV-2 infection. In the infection group, the association of prophylactic oral arbidol with hospitalization and the development of severe pneumonia was assessed.

### Statistical Analysis

The statistical software SPSS 23.0 was used in this study. The single-sample k-s test was used to test the normality of the data. Categorical variables were described as frequency rates and percentages, and continuous variables were described using mean or median values and interquartile range (IQR). Means for continuous variables were compared using independent group *t*-tests when the data were normally distributed; otherwise, the Mann–Whitney test was used. Proportions for categorical variables were compared using the χ2 test, although Fisher's exact test was used when the data were limited. Infection-free survival rates were compared using the log-rank test. Tests were performed at α = 0.05 level (both sides), and *P* < 0.05 indicates that the difference is statistically significant.

## Results

**Figure 1:**
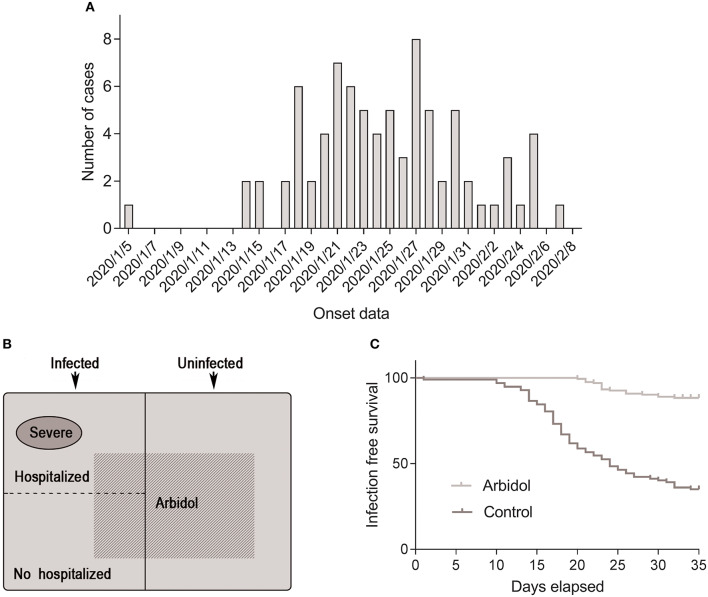
Distribution of health professionals involved in the investigation. **(A)** Onset of illness among confirmed cases of COVID-19 in Tongji Hospital. **(B)** Composition of individuals involved in this study. **(C)** Infection-free survival rate of people taking arbidol and controls over 45 days of the COVID-19 outbreak.

Since the outbreak in Wuhan, the number of confirmed cases has increased rapidly, with an initial estimated *R*_0_ of 2.2 (95% CI 1.4–3.9) (8). Similarly, the number of confirmed cases among medical personnel has continued to rise. A total of 164 people were included in this study, 82 cases in the infected group and 82 controls in the uninfected group, with a median age of 37 years, including 63 males and 101 females (Table 1). Sixty health professionals worked in high-risk departments, and 104 cases worked in non-high-risk departments. A small number of cases were accompanied by underlying diseases, mostly hypertension and diabetes. The distribution of illness onset among health professionals in the infected group included in the study is shown in Figures 1A,B.

**Table 1:** Baseline characteristics of health professionals included in the study.

**Research**	**All subjects**	**Infected**	**Uninfected**	***P*-value**
**factors**	**(*n* = 164)**	**group**	**group**	
		**(*n* = 82)**	**(*n* = 82)**	
Age, years	37 (31–46)	37 (31–46)	37 (32–43)	0.958
Department				1.000
High-risk department	60 (37%)	30 (37%)	30 (37%)	
Non-high-risk department	104 (63%)	52 (63%)	52 (63%)	
Sex				0.077
Male	63 (38%)	26 (32%)	37 (45%)	
Female	101 (62%)	56 (68%)	45 (55%)	
Occupation				0.254
Doctor	64 (39%)	27 (33%)	37 (45%)	
Nurse	85 (52%)	46 (56%)	39 (48%)	
Other	15 (9%)	9 (11%)	6 (7%)	
Any comorbidities				0.773
Yes	13 (8%)	7 (9%)	6 (7%)	
No	151 (92%)	75 (91%)	76 (93%)	
Arbidol				<0.001
Yes	67 (41%)	19 (23%)	48 (59%)	
No	97 (59%)	63 (77%)	34 (41%)	

Nineteen (23.2%) patients in the infected group were administered oral arbidol prophylactically, and 48 (58.5%) patients in the uninfected group took arbidol. A comparative analysis of the infected and non-infected groups showed that there was no correlation between SARS-CoV-2 infection and the sex, occupation, and comorbidities of health professionals, but there was a significant correlation with arbidol (23.2 vs. 58.5%, OR = 0.214, 95% CI 0.109–0.420; *P* < 0.001), indicating that arbidol is protective against COVID-19 in health professionals (Table 1). The cumulative number of COVID-19 of health professionals in our hospital continued to increase from Jan 5, 2020 to Feb 8, 2020. Insufficient protection awareness and insufficient medical protective supplies were important reasons for medical staff infection in the early stage. The cumulative uninfected rate of health professionals in the arbidol group was significantly higher than that of individuals in the non-arbidol group (log-rank test, χ^2^ = 98.74; *P* < 0.001) (Figure 1C).

Forty-eight patients (58.5%) in the infection group were hospitalized, with a median age of 39 (31–49) years, of whom 7 (14.6%) took arbidol prophylactically. Thirty-four individuals (41.5%) had mild symptoms and were isolated outside the hospital (at home or a hotel). The median age was 34 (30–39) years, and twelve individuals (35.3%) were administered oral arbidol. Among patients infected with SARS-CoV-2, a comparison analysis between the hospitalized group and the non-hospitalized group showed that hospitalization rate was associated with age (*P* = 0.024) and oral arbidol use (OR = 0.313, 95% CI 0.108–0.909; *P* = 0.029) (Table 2). Moreover, oral arbidol was also negatively correlated with duration of positive throat swab (*r* = −0.286, *P* = 0.011). Meanwhile, there was no correlation with the health professionals' sex, occupation, or comorbidities, suggesting that younger age and prophylactic oral arbidol use may protective against disease progression.

**Table 2:** Characteristics of infected health professionals included in the study.

**Research factors**	**All patients (*n* = 82)**	**Hospitalization (*n* = 48)**	**No hospitalization (*n* = 34)**	***P*-value**
Age, years	37 (31–46)	39 (31–49)	34 (30–39)	0.024
Department				0.503
High-risk department	30 (37%)	19 (40%)	11 (32%)	
Non-high-risk department	52 (63%)	29 (60%)	23 (68%)	
Sex				0.180
Male	26 (32%)	18 (38%)	8 (24%)	
Female	56 (68%)	30 (63%)	26 (76%)	
Any comorbidities				0.230
Yes	7 (9%)	6 (13%)	1 (3%)	
No	75 (91%)	42 (87%)	33 (97%)	
Arbidol				0.029
Yes	19 (23%)	7 (15%)	12 (35%)	
No	63 (77%)	41 (85%)	22 (65%)	
Neutrophils, × 10^9^ per L	3.5 (2.2–4.3)	3.3 (2.0–3.9)	4.1 (3.2–5.1)	0.242
Lymphocytes, × 10^9^ per L	1.3 (1.0–1.6)	1.2 (0.8–1.5)	1.6 (1.1–2.1)	0.011
Monocytes, × 10^9^ per L	0.5 (0.4–0.6)	0.5 (0.4–0.6)	0.5 (0.4–0.6)	0.357
Eosinophils, × 10^9^ per L	0.1 (0.0–0.1)	0.1 (0.0–0.1)	0.0 (0.0–0.1)	0.999
Basophils, × 10^9^ per L	0.0 (0.0–0.0)	0.0 (0.0–0.0)	0.0 (0.0–0.0)	0.536
Platelets, × 10^9^ per L	202.6 (160.0–235.0)	191.7 (156.0–215.0)	195.0 (158.0–217.0)	0.008
Hemoglobin, g/L	135.1 (126.0–145.0)	136.7 (126.0–148.0)	129.0 (117.8–141.0)	0.103
ALT, U/L	24.5 (11.0–29.0)	22.5 (12.0–27.0)	32.1 (10.3–55.0)	0.149
AST, U/L	26.8 (18.0–29.0)	26.1 (19.0–29.0)	29.0 (17.3–41.8)	0.932
Albumin, g/L	40.9 (38.9–44.7)	39.8 (38.5–43.5)	45.2 (44.1–48.0)	0.001
Total bilirubin, μmol/L	8.3 (5.1–9.7)	8.8 (5.1–10.1)	6.4 (4.8–7.6)	0.141
LDH, U/L	231.0 (180.8–263.0)	234.4 (184.0–266.0)	216.6 (174.0–244.0)	0.446
BUN, mmol/L	3.8 (2.9–4.5)	4.0 (2.9–4.5)	3.1 (2.7–3.7)	0.063
Creatinine, μmol/L	68.0 (55.5–78.0)	71.0 (56.0–83.0)	55.1 (50.0–59.0)	0.008
Prothrombin time, seconds	13.5 (12.9–14.0)	13.6 (12.9–14.0)	13.1 (12.7–13.4)	0.265
D-dimer, μg/ml	0.4 (0.0–0.6)	0.4 (0.2–0.5)	0.3 (0.0–0.7)	0.551
Positive throat swab, days	8 (6–12)	9 (6–14)	7 (5–11)	0.018

*Laboratory parameters were tested in 59 patients (47 cases hospitalized, 12 cases not hospitalized). ALT, Alanine aminotransferase; AST, Aspartate aminotransferase; LDH, Lactate dehydrogenase; BUN, Blood urea nitrogen*.

To minimize potential confounding effects of age, a matched case-control study was performed. However, in the age-matched case-control study, the hospitalization rate was not significantly associated with arbidol administration (*P* = 0.091) (Table 3). Furthermore, oral arbidol was not significantly correlated with duration of positive throat swab (*r* = −0.240, *P* = 0.056) when matched by age, indicating prophylactic oral arbidol might not delay of the progression of COVID-19. Four of the 48 hospitalized patients progressed to severe pneumonia, with a median age of 51 (43–62) years, all of whom had no prophylactic oral arbidol use. The median age of 44 non-critically ill inpatients was 39 (30–48) years, and 7 were administered oral arbidol. Severe pneumonia was related to age (*P* = 0.027), but no correlation was found with health professionals' sex, occupation, comorbidities, or oral arbidol use, suggesting that elderly patients were vulnerable to severe pneumonia. One of the 82 cases died of respiratory failure during hospitalization, the remaining patients were cured.

**Table 3:** Clinical characteristics of infected health professionals in the matched case-control study.

**Research factors**	**All patients**	**Hospitalization**	**No hospitalization**	***P*-value**
	**(*n* = 68)**	**(*n* = 34)**	**(*n* = 34)**	
Age, years	34.5 (30–40)	36.5 (30–41)	34 (30–39)	0.963
Department				0.318
High-risk department	26 (38%)	15 (44%)	11 (32%)	
Non-high-risk	42 (62%)	19 (56%)	23 (68%)	
Sex				0.417
Male	19 (28%)	11 (32%)	8 (24%)	
Female	49 (72%)	23 (68%)	26 (76%)	
Any comorbidities				0.500
Yes	3 (4%)	2 (6%)	1 (3%)	
No	65 (96%)	32 (94%)	33 (97%)	
Arbidol				0.091
Yes	17 (25%)	5 (15%)	12 (35%)	
No	51 (75%)	29 (85%)	22 (65%)	
Neutrophils, × 10^9^ per L	3.3 (2.1–4.2)	2.8 (1.9–3.8)	4.1 (3.2–5.1)	0.017
Lymphocytes, × 10^9^ per L	1.3 (1.0–1.6)	1.2 (0.9–1.6)	1.6 (1.1–2.1)	0.066
Monocytes, × 10^9^ per L	0.5 (0.4–0.7)	0.5 (0.4–0.7)	0.5 (0.4–0.6)	0.553
Eosinophils, × 10^9^ per L	0.1 (0.0–0.1)	0.1 (0.0–0.1)	0.0 (0.0–0.1)	0.772
Basophils, × 10^9^ per L	0.0 (0.0–0.0)	0.0 (0.0–0.0)	0.0 (0.0–0.0)	0.478
Platelets, × 10^9^ per L	208.5 (162.0–237.0)	195.0 (158.0–217.0)	195.0 (158.0–217.0)	0.052
Hemoglobin, g/L	134.0 (123.0–145.0)	136.2 (126.0–148.5)	129.0 (117.8–141.0)	0.152
ALT, U/L	22.5 (11.0–24.5)	19.1 (11.0–22.0)	32.1 (10.3–55.0)	0.639
AST, U/L	25.7 (17.5–28.5)	24.4 (18.0–28.0)	29.0 (17.3–41.8)	0.830
Albumin, g/L	41.7 (39.2–44.9)	40.5 (38.8–43.7)	45.2 (44.1–48.0)	0.001
Total bilirubin, μmol/L	8.1 (4.9–10.1)	8.8 (4.9–10.5)	6.4 (4.8–7.6)	0.502
LDH, U/L	227.4 (185.0–253.8)	230.9 (186.0–259.0)	216.6 (174.0–244.0)	0.522
BUN, mmol/L	3.6 (2.8–4.4)	3.8 (2.9–4.5)	3.1 (2.7–3.7)	0.113
Creatinine, μmol/L	65.2 (52.5–74.5)	68.6 (56.5–78.0)	55.1 (50.0–59.0)	0.025
Prothrombin time, seconds	13.6 (12.9–14.2)	13.6 (13.0–14.4)	13.1 (12.7–13.4)	0.187
D-dimer, μg/ml	0.3 (0.0–0.5)	0.3 (0.0–0.5)	0.3 (0.0–0.7)	0.668
Positive throat swab, days	8 (6–11)	9 (6–12)	7 (5–11)	0.286

*Laboratory parameters were tested in 45 patients (33 cases hospitalized, 12 cases not hospitalized). ALT, Alanine aminotransferase; AST, Aspartate aminotransferase; LDH, Lactate dehydrogenase; BUN, Blood urea nitrogen*.

## Discussion

To overcome the current severe epidemic situation, COVID-19 has become a research hotspot. At present, a large amount of literature reports the epidemiology, clinical characteristics and prognosis of the disease (1, 4, 9). However, there is no research on drug-based prevention for this special group of health professionals.

This study found that preventative oral arbidol was significantly associated with reduced SARS-CoV-2 infection rate of health professionals, which showed that arbidol might play a preventative role in health professionals. Arbidol is a broad-spectrum antiviral compound that blocks the contact, adhesion and fusion of viral lipid capsules and host cell membranes and blocks the virus replication (6, 10). *In vivo* and *in vitro* experiments confirm that arbidol has inhibitory effects on a variety of respiratory viruses, including enveloped and unenveloped viruses as well as RNA and DNA viruses (11). A randomized controlled trial gave oral arbidol (200 mg/d) to workers during an influenza epidemic for 10 to 18 days and found that arbidol had significant preventative effects (12). Similarly, Titova et al. administered oral arbidol to asthma and chronic obstructive pulmonary disease (COPD) patients to prevent viral infections (13). Recently, oral arbidol use indicated favorable clinical response in patients with COVID-19 (14). These findings are consistent with the results obtained in our study that arbidol was negatively associated with SARS-CoV-2 infection.

It is worth noting that this study found preventative oral arbidol was not significantly associated with the hospitalization rate and duration of positive throat swab of health professionals with COVID-19. Moreover, no statistical correlation between prophylactic medication and severe pneumonia, which was worth further consideration. The possible reasons were speculated as follows. Arbidol effectively block the virus from entering host cells and block the initial stages of the virus's pathogenic process, leading to preventative protection (10, 11). However, when a large number of viruses replicate in host cells, the protective effect of arbidol is limited. Therefore, combined usage of arbidol and other antiviral drugs may be a promising option. It should be noted that preventative oral arbidol was more common among non-hospitalized patients (35 vs. 15%), although this difference was not significant after matching with age (*P* = 0.091). Further studies are needed to ascertain the role and mechanism of arbidol in SARS-CoV-2 infections.

Arbidol was approved to market in China in 2006 for the treatment of upper respiratory tract infections caused by influenza A and B viruses. It is well-tolerated and safe in humans. Sixty-seven health professionals who took oral arbidol could tolerate it (6.7 days on average) in our study, among whom few people had mild diarrhea even at a therapeutic dose (~10%). No serious adverse events related to oral arbidol use have been reported.

### Limitations of This Study

This study also has limitations. It is a single-center retrospective study with a limited size and lacks a multi-center prospective cohort study for improved validation. In addition, there is no guarantee that the participant's protection awareness and protection measures were completely consistent.

### Conclusion

In summary, arbidol was significantly associated with reduced SARS-CoV-2 infection and might play a preventative role among health professionals. This conclusion also has certain significance for other high-risk populations, such as family members of COVID-19 patients and infectious disease control personnel.

## Data Availability Statement

The raw data supporting the conclusions of this article will be made available by the authors, without undue reservation.

## Ethics Statement

The studies involving human participants were reviewed and approved by The Medical Ethical Committee of Tongji Hospital of Huazhong University of Science and Technology. Written informed consent for participation was not required for this study in accordance with the national legislation and the institutional requirements.

## Author Contributions

CY, WLi, and ZH made substantial contributions to the study design. CK and DY was in charge of the manuscript draft. JL took responsibility for obtaining ethical approval. WLiu and SH took responsibility for data acquisition. CY and ZH made main contributions to data analysis and interpretation. CY and JL participated in the diagnosis and treatment of health professionals. SW made substantial revisions to the manuscript.

## Conflict of Interest

The authors declare that the research was conducted in the absence of any commercial or financial relationships that could be construed as a potential conflict of interest.
